# Anti-Hepatocellular Carcinoma Biomolecules: Molecular Targets Insights

**DOI:** 10.3390/ijms221910774

**Published:** 2021-10-06

**Authors:** Nouf Juaid, Amr Amin, Ali Abdalla, Kevin Reese, Zaenah Alamri, Mohamed Moulay, Suzan Abdu, Nabil Miled

**Affiliations:** 1 Department of Biological Sciences, University of Jeddah, Jeddah 23445, Saudi Arabia; zzalamri@uj.edu.sa (Z.A.); sbabdou8@uj.edu.sa (S.A.); 2 Biology Department, UAE University, Al Ain 15551, United Arab Emirates; a.amin@uaeu.ac.ae; 3 The College, The University of Chicago, Chicago, IL 60637, USA; 4 Weinberg Institute for Cognitive Science, University of Michigan, Ann Arbor, MI 48109, USA; aliamr@umich.edu; 5 Department of Kinesiology, Michigan State University, East Lansing, MI 48824, USA; reesekev@msu.edu; 6 Embryonic Stem Cell Research Unit, King Fahd Medical Research Center, King Abdul Aziz University, Jeddah 22252, Saudi Arabia; moulaay.l@gmail.com; 7 Life Sciences Research Unit, Center for Sciences and Medical Research, University of Jeddah, Osfan Road, Jeddah 23445, Saudi Arabia; 8 Functional Genomics and Plant Physiology Research Unit, Higher Institute of Biotechnology Sfax, University of Sfax, BP261 Road Soukra Km4, Sfax 3038, Tunisia

**Keywords:** hepatocellular carcinoma, cancer, drug, molecular target, signaling pathway, phytochemicals

## Abstract

This report explores the available curative molecules directed against hepatocellular carcinoma (HCC). Limited efficiency as well as other drawbacks of existing molecules led to the search for promising potential alternatives. Understanding of the cell signaling mechanisms propelling carcinogenesis and driven by cell proliferation, invasion, and angiogenesis can offer valuable information for the investigation of efficient treatment strategies. The complexity of the mechanisms behind carcinogenesis inspires researchers to explore the ability of various biomolecules to target specific pathways. Natural components occurring mainly in food and medicinal plants, are considered an essential resource for discovering new and promising therapeutic molecules. Novel biomolecules normally have an advantage in terms of biosafety. They are also widely diverse and often possess potent antioxidant, anti-inflammatory, and anti-cancer properties. Based on quantitative structure–activity relationship studies, biomolecules can be used as templates for chemical modifications that improve efficiency, safety, and bioavailability. In this review, we focus on anti-HCC biomolecules that have their molecular targets partially or completely characterized as well as having anti-cancer molecular mechanisms that are fairly described.

## 1. Introduction

Being the fourth leading cause of cancer-related deaths, liver cancer continues to be a burden for the global healthcare system [1,2]. The majority (75–85%) of primary liver cancers are hepatocellular carcinoma (HCC) [3]. Besides being associated with high morbidity, HCC is the sixth leading cause of cancer related death and is characterized by a considerable phenotypic and molecular heterogeneity. Pathways involved in essential cancer related events including angiogenesis, cell proliferation, and apoptosis are main targets for HCC drug development. Tyrosine kinase receptors, Ras/Raf/mitogen-activated protein kinase (MAPK), PI3K/Akt/mTOR, Janus kinase (JAK)/Signal transducer, and activators of transcription (STAT) and Wnt/β-catenin pathways are a few examples of key signaling molecules of interest when it comes to developing an anti-cancer drug [4]. In addition, transcription factors like nuclear factor-kappa B and cell cycle regulators such as cyclins and cyclin-dependent kinases (CDKs) serve as attractive anti-HCC drug targets [4]. Angiogenesis is essential for tumor progression through the proliferation and differentiation of endothelial cells under the influence of numerous promoting factors such as cytokines and vascular endothelial growth factor (VEGF) [5]. Other growth factors such as fibroblast growth factor (FGF), angiopoietins, platelet-derived endothelial cell growth factor (PD-ECGF), hepatocyte growth factor (HGF), insulin-like growth factor (IGF), and epidermal growth factor (EGF) play crucial roles in angiogenesis and cell proliferation. Impairing angiogenesis and the binding of growth factors to their receptors are also strategies used to prevent cancer progression [5]. Apart from surgical resections or liver transplantation during early stages of HCC [6], tumor intra-arterial infusion of chemotherapeutic agents remains the conventional approach for cancer treatment [7]. Unfortunately, this approach has been reported to harm normal cells as well [8]. Efficiency of HCC treatment remains limited, and it desperately awaits a better understanding of the cancer’s molecular biology. Natural biomolecules derived mainly from plants have been widely explored for their anti-HCC and hepatoprotective effects. Thanks to their diverse biological activities (antioxidant, anti-inflammatory, anti-cancer, and immuno-modulatory) and chemical diversity, biomolecules from edible or medicinal plants are valuable sources of anti-cancer therapeutic molecules [9]. This review focuses on the most promising natural or chemically modified anti-HCC biomolecules and their molecular mechanisms of action.

## 2. HCC Targeted Therapy

### 2.1. Tyrosine Kinase Inhibitors

#### 2.1.1. Sorafenib

**Figure 1:**
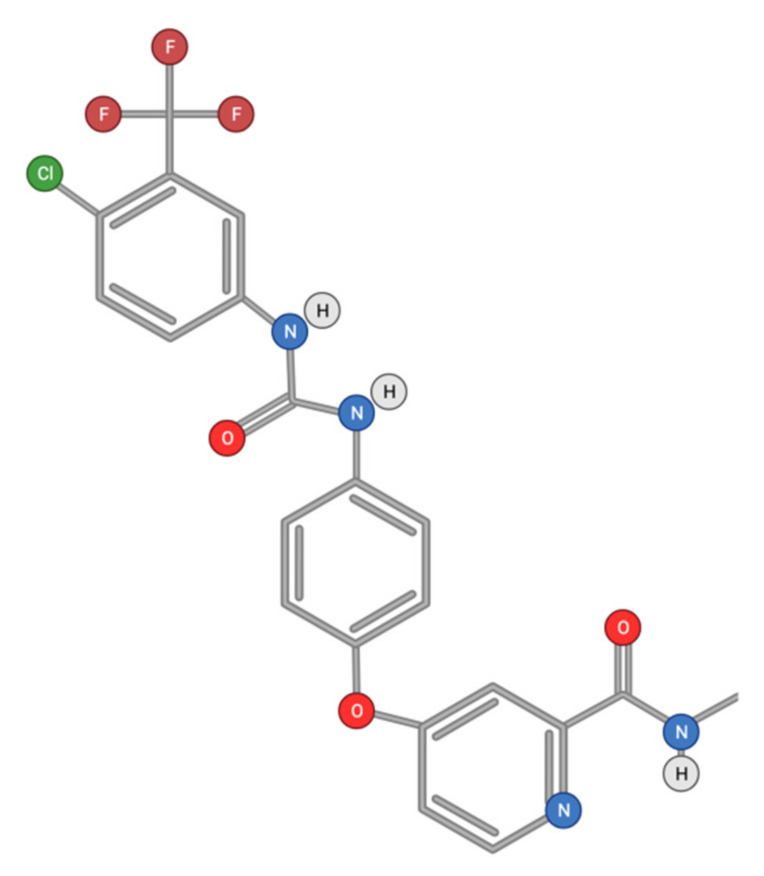
Chemical structure of Sorafenib.

Sorafenib (Figure 1) (*N*-(3-trifluoromethyl-4-chlorophenyl)-*N*′-(4-(2-methylcarbamoyl pyridin-4-yl)oxyphenyl)urea) is the result of modifications of functional groups of 3-thienyl urea used as an Raf inhibitor [10].

**Figure 2:**
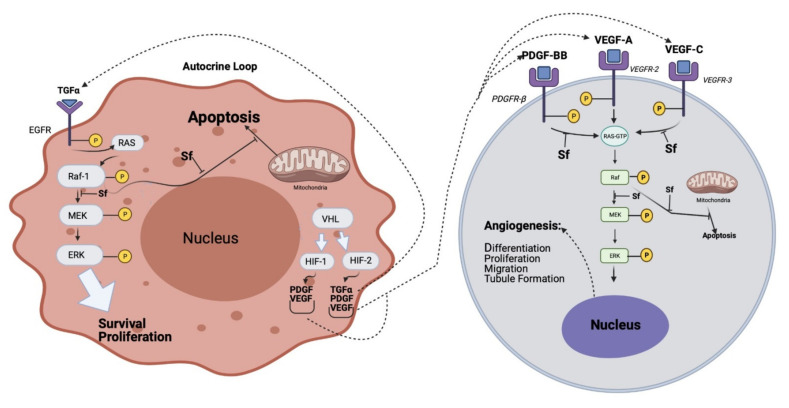
Dysregulation of Signaling through Raf-1 in tumor cells (left panel), endothelial cells, and/or pericytes (right panel) that could result in tumor growth and/or angiogenesis by an autocrine mechanism in HCC, and the effect of sorafenib (Sf), The white arrows indicate the effect on intra-cellular signal molecules or pathways. The dotted arrows indicate the effect on cell membrane receptors (Modified from [12]).

Once administered, sorafenib is metabolized mainly in the liver by cytochrome p450 oxidase (CYP3A4) as well as UGT1A9 mediated glucuronidation [11]. As a multi-kinase inhibitor, sorafenib inhibits multiple cell surface tyrosine kinases such as VEGFR, platelet-derived growth factor receptor (PDGFR)-β, and downstream MAPK serine/threonine kinases (b-RAF, c-RAF and Raf-1) (Figure 2) (Modified from [12]). These kinases are involved in the tumor cell signaling that controls proliferation, angiogenesis, and apoptosis.

Upon binding to Raf-1 sorafenib may cause disarray in Raf-1 dependent pathways, which are known to play a role in survival signaling mechanisms for cancer cells including MEK/ERK, MST-1, and ASK-1 [12]. Sorafenib has been reported to inhibit b-Raf in tumor cell lines expressing wild-type but not V600E b-Raf mutant, demonstrating a conceivable function of this residue in the interaction with sorafenib [12]. Nevertheless, sorafenib is not a universal kinase inhibitor. It did not inhibit MEK-1 or extracellular signal-regulated kinase (ERK-1) activities in vitro. Sorafenib was found to deactivate the autophosphorylation of pro-angiogenic receptors such as VEGFR and PDGFR in vitro [12]. In addition to its tyrosine kinase inhibitory effect, sorafenib was described to induce cell death through the inhibition of elF4E phosphorylation and Mcl-1 expression in tumor cells [10]. Sorafenib was also reported to reduce cancer growth, metastases, and epithelial to mesenchymal (EMT) transition by reducing the secretions of the tumor growth factor-β by tumor-associated macrophages. It was also reported that sorafenib induces tumor cell death via various MAPK-independent mechanisms, including down-regulation of anti-apoptotic Bcl-2 proteins such as myeloid cell leukemia-1 (Mcl-1) and up-regulation of p53 [10]. Interestingly, the leak of cytochrome c from the mitochondria into the cytosol, a process that triggers caspase activation and ultimately induces apoptosis, was shown to be tightly associated with sorafenib [12]. Down-regulation of Mcl-1 can be mediated by inhibiting the phosphorylation of its initiation factor, elF4E [10]. Sorafenib was also suggested to exert immunomodulatory effects through the activation of tumor-specific T cells [13].

Sorafenib was found to improve survival in patients with advanced HCC, and to display a minimal side effect profile [14]. Nevertheless, most patients who receive sorafenib eventually develop a resistance to the drug [15]. Possible drug resistance mechanisms include EMT transitions; changes in the tumor microenvironment involve angiogenesis, inflammation, fibrosis, hypoxia, oxidative stress, autophagy, and activation of an escape pathway (like PI3K/Akt/mTOR) from the MAPK cascade [16]. The drug was also proposed to upregulate hypoxia-inducible factor-2α (HIF-2α), which contributes to sorafenib’s resistance by activating the transforming growth factor (TGF)-α/EGF receptors pathway in HCC cells [15]. Resistance to sorafenib in HCC can also be driven by the activation of the Akt/β-catenin pathways [17], whereas the inhibition of the JAK/STAT pathways may overcome such resistance [18]. Combination of sorafenib with an Akt-inhibitor could reduce resistance to the treatment [19]. Combining sorafenib with mammalian targets of rapamycin (mTOR) inhibitors such as everolimus was found to be ineffective in HCC treatment [20]. Furthermore, the combination of sorafenib and ceramide might be a promising new therapeutic strategy for HCC [21]. The anti-HCC effect of sorafenib was enhanced by C2-ceramide via EMT inhibition, apoptosis induction, and cell cycle arrest. Sorafenib combined with ceramide increased the accumulation of intracellular reactive oxygen species (ROS), promoting caspase dependent cell apoptosis while restraining cell proliferation via PI3K/Akt/mTOR pathways.

**Figure 3:**
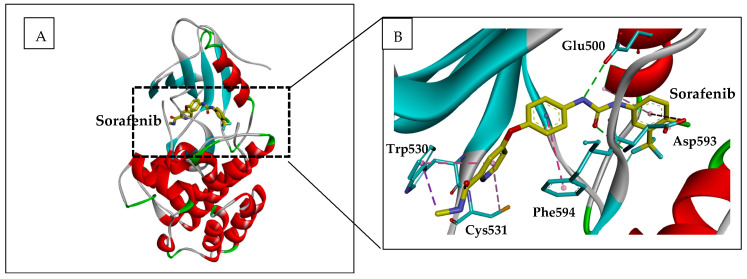
Representation of Sorafenib in complex with B-Raf. (**A**) Ribbon representation of b-Raf in complex with Sorafenib (yellow sticks). The 3-D structure used (pdb code 1UWH) was uploaded from the protein data bank (https://www.rcsb.org/structure/1UWH). (**B**) zoom from A showing interacting residues (blue sticks) with Sorafenib in the active site of b-Raf. Figure was constructed using the BIOVIA Discovery Studio software (BIOVIA, Dassault Systèmes, [BIOVIA Discovery Studio], [v17.2.0.16349], San Diego: Dassault Systèmes, [2017]).

The crystal structure of sorafenib in the active site of Raf showed that it lies between the N- and C-lobes in the catalytic cleft [1]. Interactions between the protein and the inhibitor are conserved for c-Raf and b-Raf. This explains the inhibitory effects of sorafenib derivatives on c-Raf [22]. Binding of sorafenib to the active site of b-Raf showed that the pyridyl ring is stabilized within the ATP binding pocket through hydrophobic interactions with aromatic residues Trp530, Phe582 (located at the end of the catalytic loop), and Phe594, which belongs to the DGF motif [1] (Figure 3). At the opposite site of the phenyl group, interacting with Phe582, the lipophilic trifluoromethyl phenyl may occupy a hydrophobic pocket between the catalytic loop, the αC and αE helices, and the DGF motif (Figure 3). Apart from hydrophobic contacts, the inhibitor also establishes hydrogen bonding interactions through its urea group with catalytic residue Glu500 and the NH group of Asp593, the first residue of the DGF motif. The nitrogen of the pyridyl ring was found to enhance the affinity to the active site five-fold as compared to a carbon atom [22]. The nitrogen group has hydrogen bonded to the NH group of Cys531 in the interdomain hinge region (see Figure 3).

Similar hydrogen bonding networks with catalytic residues and DGF motif were also described in the interaction of other inhibitors with Raf related kinases c-Abl and p38 [23,24]. Sorafenib analogs that contain a sulfonylurea unit were synthesized [25] and displayed an anti-proliferative effect on cancer cell lines. Structure–activity relationships and docking studies showed the important role of the sulfonylurea group in kinase inhibition. Likewise, diaryl thio-urea containing sorafenib derivatives showed anti-proliferative effects on cell lines and inhibition of EGFR phosphorylation [26]. *N*-methylpicolinamide-4-oxy chalcone sorafenib derivatives displayed cytotoxicity against HCC cell lines and an inhibitory effect on VEGFR-2/KDR and b-RAF kinases [27].

#### 2.1.2. Other Tyrosine Kinase Inhibitors

Tyrosine kinase inhibitors usually target proliferation, angiogenesis, and apoptosis. Some tyrosine kinase inhibitors are under clinical trials for HCC treatment (Table 1). Brivanib alaninate, an inhibitor of both VEGFR and FGFR tyrosine kinases, was evaluated in a phase II clinical study in patients with advanced HCC. The median overall survival was 10 months with manageable adverse events [28,29].

Moreover, Lenvatinib targets the FGF receptor (FGFR1-4), vascular endothelial growth factor receptor (VEGFR1-3), platelet-derived growth factor receptor a (PDFGRa), RET (Rearranged during Transfection), and KIT receptors. Linifanib, a tyrosine kinase inhibitor targeting VEGFR, PDGFR, and ramucirumab, was directed against VEGFR2 and failed in phase III studies in first-line and second-line indications, respectively [38,39,40]. Other molecules such as anti-angiogenic agents (vatalanib, axitinib, and cediranib), c-MET, MEK, and TGF-beta (TGFβ) inhibitors are being tested in early clinical investigations.

**Figure 4:**
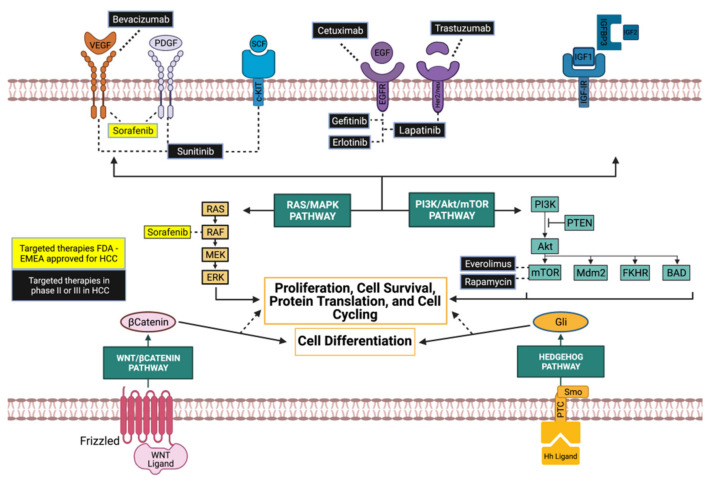
Molecular targeted therapies in HCC. Tyrosine kinase and mTOR inhibitors in preclinical studies or clinical trials for HCC. Some monoclonal antibodies directed against tyrosine kinase receptors are also indicated (Modified from [47]).

The effects of sorafenib and other tyrosine kinase inhibitors on major cancer related signaling pathways are in phase II/III clinical trials and are shown in Figure 4.

**Figure 5:**
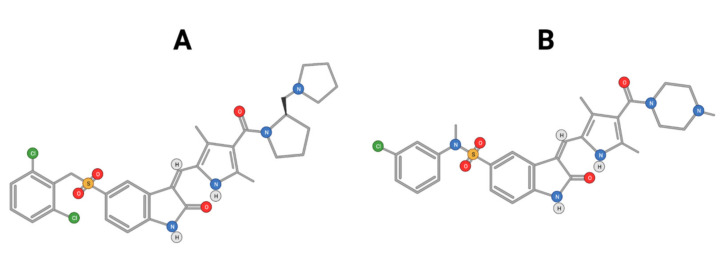
Chemical structure of PHA665752 (**A**) and SU11274 (**B**) compounds.

Cabozantinib, an inhibitor of MET, VEGFR2, and RET is approved for thyroid and renal cancer and is under evaluation in a Phase III randomized controlled trial for patients with advanced HCC following positive results from a Phase II study [48]. SU11274 and PHA665752 are chemically related compounds that are reported to be inhibitors of c-Met kinase activity (Figure 5) [49]. PHA665752 functions as a selective inhibitor of c-Met phosphorylation. Treatment with PHA665752 impaired c-Met phosphorylation at multiple tyrosine residues and reduced downstream phosphorylation of Akt and Erk in c-Met positive MHCC97-L and MHCC97-H cell lines [50].

### 2.2. PI3K/Akt/mTOR Inhibition

**Figure 6:**
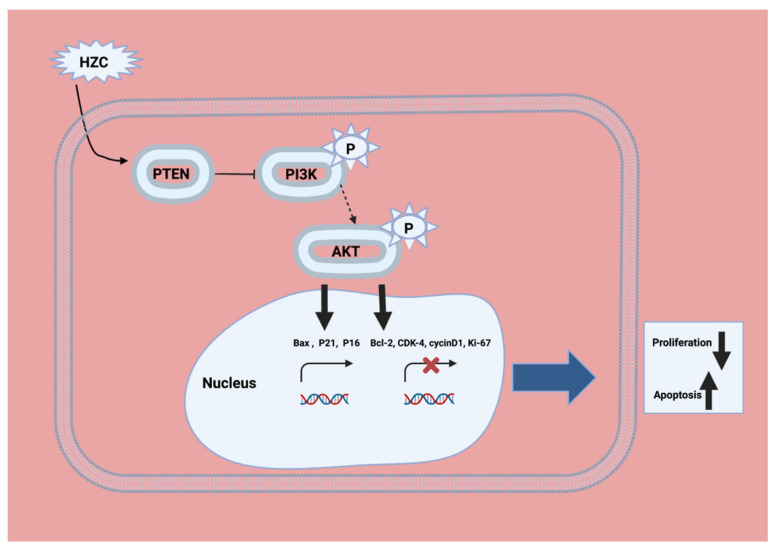
The mechanism of HZC in the HCC treatment model through the PTEN/PI3K/Akt pathway. HZC could inhibit activation of the PI3K/Akt signaling pathway by enhancing PTEN transcription, and then induce apoptosis of HepG2 cells via regulating the expression of apoptosis-related proteins (Modified from [63]).

Phosphoinositide 3-kinase (PI3K) is activated by receptor tyrosine kinases (RTKs), such as the platelet-derived growth factor receptor (PDGFR) [51]. Isoform delta of PI 3-kinase is activated by G protein coupled receptors [52]. PI3K was shown to be an activator of the proto-oncogene Akt (also named protein kinase B (PKB)) by growth factors such as PDGF [53]. The phospholipid products of PI3K, initially PI (3,4) P2, were shown to activate Akt through binding to its pleckstrin homology domain [54]. The phosphatidylinositol-3 kinase (PI3K)/Akt-pathway was activated in approximately 50% of patients with cirrhosis and HCC [55,56]. Hydrazinocurcumin (HZC) and 5-fluorouracil (5-Flu) both enhance apoptosis and restrain tumorigenicity of HepG2 cells via disruption of the PTEN-mediated PI3K/Akt signaling pathway. HZC and 5-Fu enhance the expression of PTEN, a key tumor suppressor, thus disrupting the activation of the PI3K/Akt signaling pathway (Figure 6) [57].

Adenosine MonoPhosphate (AMP)-activated protein Kinase (AMPK) is a serine/threonine kinase that can be activated through auto-phosphorylation. It plays a crucial role as a metabolic sensor and regulator of cell proliferation [58]. Mammalian target of rapamycin (mTOR) is a serine/threonine kinase that can be activated by the AMPK. It is involved in regulating cell survival, proliferation, and angiogenesis by integrating signals downstream of the PI3K/Akt pathway [59] (see Figure 4). mTOR is usually upregulated in HCC and is associated with earlier recurrence and a poor prognosis [55]. Inactivation of mTOR to restrain cancer cell proliferation was suggested as a promising strategy for the treatment of HCC.

Several preclinical studies demonstrated the effectiveness of mTOR inhibition in combating HCC. Rapamycin, a macrolide isolated from the bacterial strain Streptomyces hygroscopicus and its analogs (everolimus, temsirolimus, and ridaforolimus), was described to be effective in inhibiting mTOR [60]. Everolimus, sirolimus, and temsirolimus were also shown to inhibit proliferation of human cancer cells [61]. In HCC rodent models, everolimus significantly reduced angiogenesis and tumor growth, which improved overall survival [62]. Among the two mTOR multiprotein complexes (mTORC1 and mTORC2), these inhibitors target mTORC1 [59]. Upstream regulators of mTOR are mainly PI3K/Akt, AMPK, the phosphatase tensin homolog (PTEN), Rag guanosine triphosphatases (GTPases), and tuberous sclerosis complex (TSC) proteins. Downstream targets of mTOR include S6 kinase 1 (S6K1) and the initiation factor 4E-binding protein 1 (4E-BP1), both of which regulate the translation of proteins.

### 2.3. JAK/STAT Pathway

The JAK/STAT pathway is involved in cell proliferation, stem cell differentiation, and modulation of the immune/inflammatory response [64]. JAK/STAT signaling plays crucial roles in liver regeneration and gluconeogenesis [65]. The JAK/STAT pathway can be activated upon the binding of interleukins, interferon, and EGF family members to their receptors. The cytoplasmic tails of some of these receptors are associated with the JAKs. JAK activation triggers receptor phosphorylation which recruits transcription factors STATs. Once phosphorylated by JAK, activated STAT can bind to specific promoters and enhance the transcription of target genes, such as Mcl-1 and CCND1 [66].

**Figure 7:**
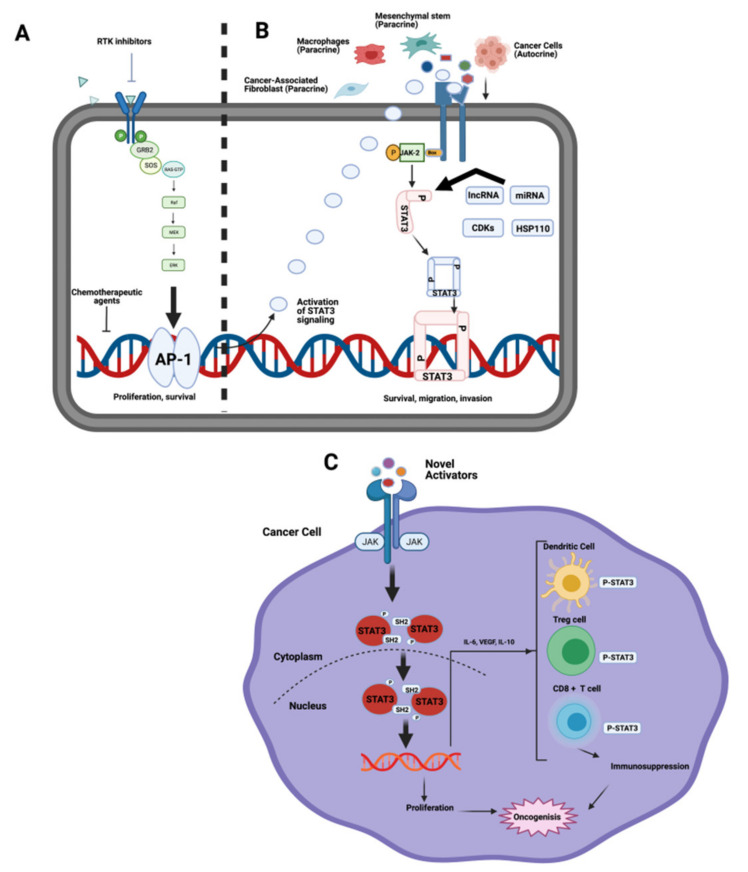
Activation of STAT3 in cancer cells. (**A**) Inhibition of cancer signaling activates STAT3 pathways. (**B**) Novel activators of STAT3 secreted by cancer cells, mesenchymal stem cells, cancer‒associated fibroblast cells, or macrophages. (**C**) Activators of STAT3 promote cancer growth through immunosuppression. STAT3 target genes IL-6, IL-10, and VEGF are regulated by STAT3. These tumor-associated factors activate STAT3 in the immune system (Modified from [72]).

JAK is a target to inactivate the downstream JAK/STAT pathway (Figure 7). JAK2, STAT5A, and STAT6 were described as potential prognostic biomarkers for HCC [67]. Meanwhile, TYK2, STAT3, STAT4, and STAT5B can be used as diagnostic biomarkers for HCC. Ras and JAK/STAT pathway activation is enhanced in HCC cells as compared to non-neoplastic normal liver cells. Concomitantly, Ras (NORE1A and/or RASSF1A) and JAK/STAT inhibitors (SH2-containing phosphatase, a suppressor of cytokine signaling (SOCS) and cytokine-inducible SH2-protein (CIS)) were blocked. HCC associated with cirrhosis displayed a significantly higher frequency of CIS, SOCS1, and RASSF1A promoter methylation [68]. Furthermore, aberrant methylation of SOCS3 and NORE1A promoters were observed in an HCC subclass with poor survival, suggesting that suppression of these two genes might be involved in HCC progression. Interestingly, treatment of HCC cell lines using a combination of Ras and JAK/STAT inhibitors as well as a demethylating agent (zebularine) promoted a strong apoptotic response. WP1066, pacritinib, cryptotanshinone, and ruxolitinib are examples of JAK inhibitors being studied in preclinical phase [69]. They were shown to reduce both cancer cell proliferation and invasion. Pacritinib was found to reduce liver fibrosis in mouse models that mimic clinical HCC development, since fibrosis occurs in most HCC patients. Ruxolitinib was shown to exert an anti-proliferative effect on HCC cells, and to inactivate JAK/STAT signaling via reducing the expression of phosphorylated STAT (pSTAT1 and pSTAT3). Six-hydroxy-3-*O*-methyl-kaempferol 6-*O*-glucopyranoside enhances the anti-proliferative effect of interferon α/β by regulating JAK/STAT signaling via suppressing SOCS3 in HCC cells [70]. HCC is a typical example of an inflammation related cancer where increased IL-6 and TNFα may be responsible for the activation of JAK/STAT3 signaling [71] (Figure 7). Activators of STAT3 promote cancer growth through immuno-suppression. STAT3 target genes, VEGF, IL-10, and IL-6 are transcriptionally regulated by STAT3 and are propagated from cancer cells to immune cells. These tumor-associated factors then activate STAT3 in the immune system [72]. Moreover, Stattic is a multi-kinase inhibitor targeting STAT3 in the preclinical phase that shows the potential to reduce cancer cell survival, proliferation, and invasiveness [73].

## 3. Micro RNAs (miRNAs) in HCC Treatment

Micro-RNAs (miRNAs) are small non-coding RNAs made of 19–22 Nucleotides [74] which regulate the transcriptional process. They bind to the 3′ UTR end of pre-mRNA, which leads to degradation. miRNAs are transcribed by RNA polymerase II before being formed through Drosha processing in the nucleus which is then followed by exportation and Dicer cleavage in the cytosol [75]. miRNAs were often described in malignant tumors and are receiving increased attention as a viable alternative for cancer treatment. Exosomes, which are small membrane-bound vesicles involved in cell–cell communication, were described to be carriers for miRNAs [76]. Exosomes carry various cargos from a donor cell to an acceptor one including proteins, DNA, RNA fragments, and miRNAs. In addition to their potential role as HCC biomarkers, the possible therapeutic capacity of miRNAs has also been reported in HCC treatment [76].

**Figure 8:**
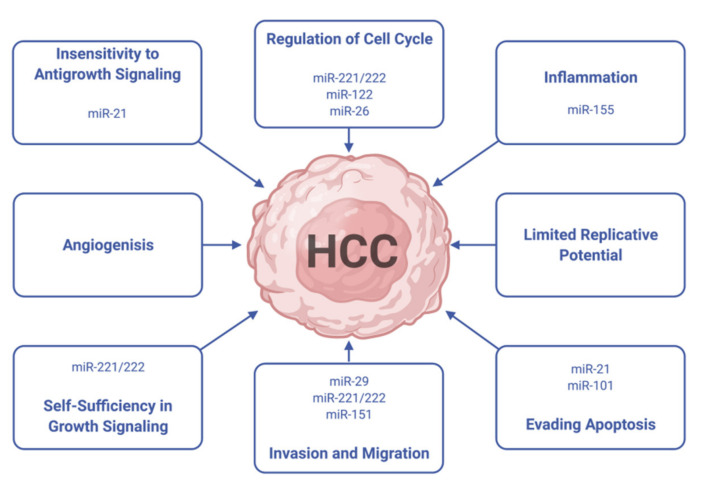
Several miRNAs act synergistically to promote HCC through the modulation of multiple cell phenotypes (Modified from [77]).

Tumor-suppressing or aberrantly expressed targeted miRNAs can be a powerful and highly specific anticancer therapeutic modality for HCC [77]. miRNAs are involved in all cancer related pathways including but not limited to cell proliferation, apoptosis, and angiogenesis (Figure 8).

The way miRNAs are involved in HCC progression and metastasis is still being investigated. Both normal and cancer cells can take up exosomal miRNAs from the extracellular fluids where they can employ various regulative functions. Cancer cell exosomes were described to selectively concentrate miRNAs [78]. Some miRNAs such as miR-584, miR-517c, and miR-378 can enhance hepatocarcinogenesis if transferred from cancer cells to recipient normal cells. Upon transferring into the tumor microenvironment, the exosomal miRNAs promote cancer cell growth through controlling the expression of the TGF-β-activated kinase-1 (TAK1) in recipient HCC cells [79]. Exosomal miRNAs can therefore play a promoting role in intra-hepatic or multi-focal metastases associated with HCC. miRNAs are also shown to be involved in inflammation. miRNA-155 was described to be transferred from chemically induced inflamed liver cells, to normal liver cells by exosomes [74]. This could lead to a pro-inflammatory phenotype, associated with increasing levels of interleukins (IL-6 and IL-8) that are involved in carcinogenesis, through the activation of the p-STAT3 pathway.

Exosomal miRNAs can inhibit HCC progression, and an advanced clinical stage of HCC was correlated with a reduction in miR-451 yields [80]. The miR-451 was demonstrated to inhibit cell growth, induce G0/G1 arrest, and promote apoptosis in HCC cells. miRNA-122 is the most abundant miRNA in the liver and is specific to hepatic cells. It is described as a tumor suppressor in HCC [81]. miRNA-122 was shown to be transferred through exosomes from Huh7 to HepG2 liver cells in a co-culture and acted as an anti-cancer agent by inducing apoptosis [82]. Interestingly, cancer cells were able to down regulate the expression of miRNA-122 in normal cells by using the secretion of insulin like growth factor 1. This is an example of the regulation of cancer progression by the cell microenvironment. Baculovirus (BV) vectors that expressed miR-122 precursors (pre-miR-122) were found to combat HCC tumorigenicity and metastasis [83]. In fact, transduction of aggressive HCC cells with the pre-miR-122-expressing BV enhanced miR-122 levels and suppressed levels of downstream effectors (ADAM10 and Bcl-w), proliferation, migration, and invasion of tumor cells. Exosomal miR-335-5p and 320 are also distinctive promising therapeutic agents. Exosomal miR-335-5p secreted by liver LEX2 cells was also reported to inhibit the proliferation of HCC cells [84]. Moreover, miRNA-9-3p was described to act as an anti-HCC agent through repression of the proliferative fibroblast factor gene [85]. Through binding to its downstream regulator PBX3, miR-320a suppressed HCC cell proliferation and metastasis [51]. The miR-320a-PBX3 pathway inhibited tumor progression through suppression of the activation of the MAPK pathway, downregulating the cyclin-dependent kinase 2 (CDK2), and MMP2 expression. Furthermore, overexpression of miRNA-320a in cancer-associated fibroblasts used in a xenograft model inhibited tumorigenesis. Cross-talking between exosomal signaling and hepatitis virus in the cancer cells were also recently described. Following the stimulation by hepatitis C virus E2 envelope glycoprotein, mast cells were able to transfer exosomal miR-490 into HCC cells, which blocked their growth. This inhibition was explained by targeting the ERK1/2 pathway in the HCC cells [86]. The therapeutic value of a miRNA replacement strategy was described for miR-26a, a miRNA whose expression is frequently lost in HCC. miRNA-26 was shown to improve chemosensitivity and induce apoptosis in HCC cells through autophagy [87]. miR-26a can target cyclins D2 and E2 and induce G1 arrest when expressed in human liver cancer cells.

## 4. Phytochemicals as Potential Anti-HCC Biomolecules

### 4.1. Polyphenols

#### 4.1.1. Phenolic Compounds

**Figure 9:**
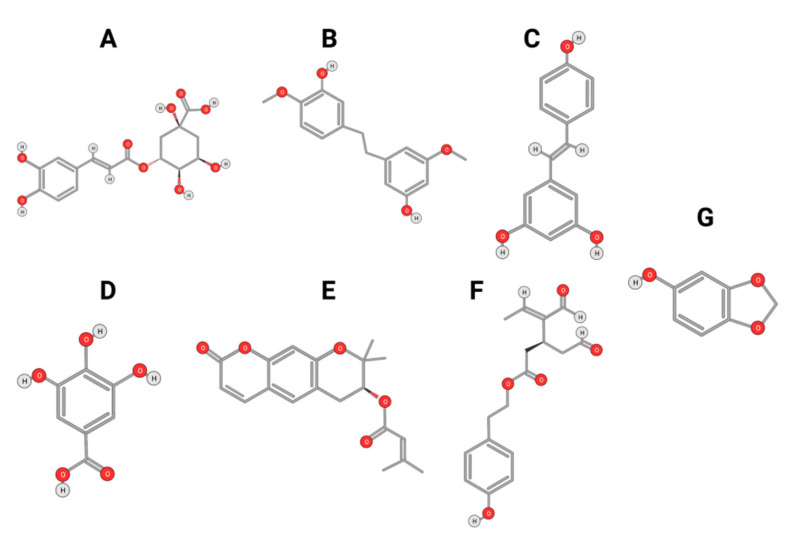
Chlorogenic Acid (**A**); Gigantol (**B**); Resveratrol (**C**); Gallic Acid (**D**); Decursin (**E**); Oleocanthal (**F**); and Sesamol (**G**).

In addition to their well-known antioxidant effect, many polyphenols were described as displaying anti-HCC activity through targeting intra-cellular signaling pathways. Curcumin is a polyphenolic compound that can be found in the ginger family. It was described as displaying anti-carcinogenic activity by suppressing the expression of glypican-3 and VEGF [88]. Similarly, anti-proliferative activity against HepG2 cells was reported for chlorogenic and Gallic acids (Figure 9A,B).

The Chlorogenic acid anti-HCC activity was explained by an inactivation of ERK1/2, and the suppression of MMP-2 and MMP-9 expression in both HepG2 and xenograft animal models [89]. Similarly, gallic acid was proven to be a potent anti-proliferative agent against DEN-induced HCC in rats [90]. Gigantol, (Figure 9C) a bibenzyl compound isolated from Dendrobium orchids, was also found to exert an anti-proliferative effect on HepG2 cells by regulating PI3K/Akt/NF-κB signaling pathways [91].

Decursin (Figure 9D), a major compound isolated from the root of the Korean Dang-gui (Angelica gigas Nakai, AGN), inhibited the growth of HepG2 cells [67]. Decursin provoked apoptosis that could be reversed by a selective MST1/2 inhibitor. This suggested that decursin also acted through Hippo-Yes-Associated Protein signaling. On both HepG2 cells and xenograft models, resveratrol (Figure 9E) extracted from grapes displayed an anti-HCC effect explained by a reduction of MMP-9 expression via downregulation of Nuclear Factor κB (NF-κB) and HGF-c-Met pathways [92]. A phenolic compound extracted from virgin olive oil (Oleocanthal, Figure 9F) was described as displaying anti-HCC activity in an orthotopic HCC animal model [93]. This activity was triggered by the inhibition of STAT3 by decreasing the expression of JAK1 and JAK2 and enhancing SHP-1 levels. Likewise, sesamol (Figure 9G), a phenolic compound found in sesame seeds, can suppress colony formation, inhibit proliferation, and promote apoptosis regulation of the PI3K class III/Belin-1 pathway in HepG2 cells and in a xenograft nude mice model [94].

#### 4.1.2. Flavonoids

##### Quercetin

**Figure 10:**
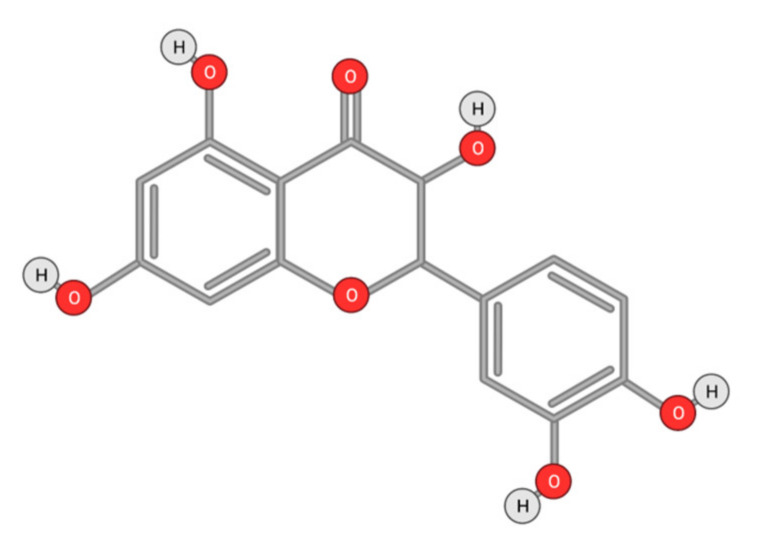
Chemical structure of quercetin.

Quercetin (Figure 10) is a plant flavonol contained in many plants including onions, broccoli, raspberries, apples, citrus, Nelumbo nucifera, and leafy greens. Quercetin is chemically related to luteolin (with an additional OH group). It inhibits angiogenesis factor VEGF’s expression via multiple signaling pathways, including Akt/mTOR, and via interaction with NF-KB nuclear transcription protein [9].

**Figure 11:**
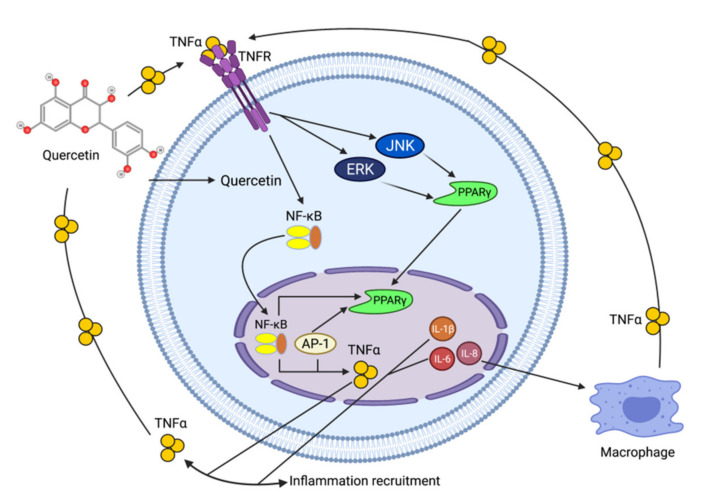
Working model on how quercetin blocks tumor necrosis factor-α (TNFα)‒mediated inflammation (Modified from [98]).

Quercetin was able to regulate the functions of phosphatidylinositol (PI)-3-kinase, PKC, COX-2 and inducible nitric oxide synthase (iNOS), causing an upregulation of pro-apoptotic p53 and BAX in HepG2 cells [95,96,97]. The anti-cancer and hepatoprotective effects of quercetin that is widely reported might be partly driven by its anti-inflammatory effect (Figure 11). Quercetin stops TNF-α from inducing extracellular signal-related kinase (ERK), c-Jun NH2-terminal kinase (JNK), and NF-κB, which are prominent activators of inflammation. Furthermore, quercetin may indirectly block inflammation by inducing peroxisome proliferator-activated receptor c (PPARγ) activity, thereby antagonizing NF-κB and/or inflammatory genes’ transcriptional activator protein (AP-1). This leads to the inactivation of TNF-α-mediated inflammatory cascades [98].

##### Other Flavonoids

**Figure 12:**
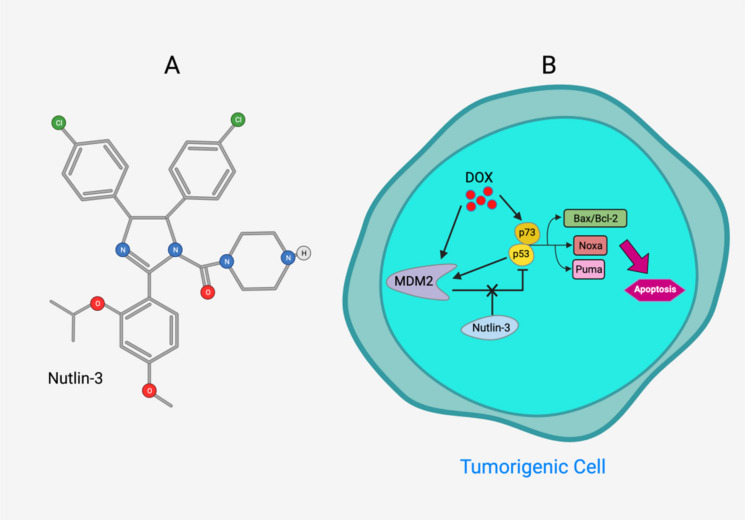
(**A**) Chemical structure of Nutlin‒3. (**B**) Nutlin‒3 inhibits binding of p53 and p73 to MDM2 when combined with DOX and increases p53 and p73 activity in human HCC cell lines (Modified from [109]).

Tea flavanols displayed an anti-carcinogenic effect through the regulation of self-renewal Wnt/beta-catenin pathways, and their associated genes cyclin D1, cMyc, and EGFR, along with the downregulation of E-cadherin [99]. Oroxin B, a flavone glycoside isolated from Oroxylum indicum, inhibited the proliferation of liver cancer cells by suppressing VEGF and PTEN/PI3K/Akt signaling pathways in addition to inactivating Cox2 [100]. Similarly, Scutellarein, a flavone present in S. lateriflora, reduces oxidative stress and suppresses the development of liver cancer by impairing the expression of VEGFA, Flt-1, HIF-1α, MMP2, and MMP9. It also induces apoptosis by promoting caspase-3 controlled nucleosomal degradation [101]. Ilexgenin A, an active pentacyclic triterpenoid component derived from Ilex hainanensis is used as a traditional remedy for curing dyslipidemia, inflammation, and hypertension [102]. In HepG2 cells, ilexgenin A exerts antitumor activity by promotion of cell cycle arrest. It inhibits VEGF production and transcription, inhibits the PI3K and STAT3 signaling pathways, and suppresses the inflammatory cytokines IL-6 and TNF-β [102]. Likewise, a flavonoid constituent derived from P. villosa (Apigenin) inhibited HCC cell growth by inducing G1 phase arrest in HepG2 cells through the activation of the p38 MAPK-p21 signaling pathway and the regulation of cyclin D1-CDK4 complex formation [103]. Moreover, delphinidin was described to inhibit epidermal growth factor induced EMT transition in hepatocellular carcinoma cells [104]. Safranal, a terpenoid derived from Saffron, showed prominent preventive [105,106] and therapeutic [107] anti-HCC effects. It induced cell cycle arrest and damage of DNA machinery. It also displayed a pro-apoptotic effect through caspase activation and endoplasmic reticulum stress-induction [108]. Through the screening of a chemical library, a cis-imidazoline analog (Nutlin-3, Figure 12) was found to disrupt p53–MDM2 binding (in HepG2 cells) and p73–MDM2 (in Huh-7 and Hep3B cell lines), leading to the activation of doxorubicin (DOX)-induced apoptosis [109].

The origins, mechanisms, and actions of other flavonoids are summarized in Table 2. It is noteworthy that the anti-HCC activity of flavonoids is mainly driven by the inhibition of cell proliferation and angiogenesis, as well as the induction of apoptosis. The anti-HCC effects of many flavonoids such as epigallocatechin gallate [110], theaflavins [111] (tea leaves), hesperidin [112] (citrus fruit), and kurarinol [113] were demonstrated both in vitro and in vivo. These compounds inhibit cancer cell growth by targeting key growth signaling cascades such as tyrosine kinase receptor, Ras/MEK/ERK, and PI3-kinase Akt/mTOR pathways. They also promote apoptosis through the activation of the pro-apoptotic p53, Bcl-2, and caspace-9 mediators.

### 4.2. Carotenoids and Alkaloids

**Figure 13:**
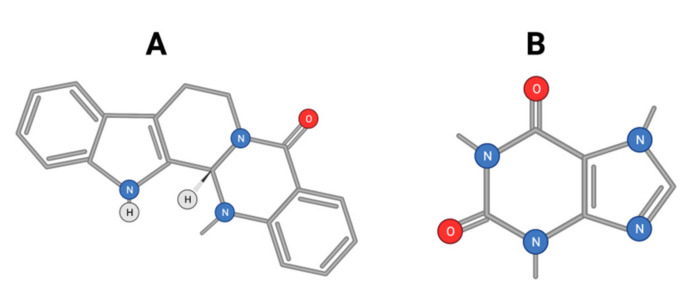
Chemical structure of evodiamine (**A**) and caffeine (**B**).

Alkaloids were described to be effective in inhibiting HCC development [131]. Evodiamine (Figure 13A) is a major alkaloid isolated from the Chinese herbal medicine Evodia rutaecarpa. Evodiamine exerts anti-tumor effects against HCC by inhibiting β-catenin-mediated angiogenesis [132].

Additionally, evodiamine was shown to induce the apoptosis of HCC through downregulating HIF-1α under hypoxia [133]. It was also reported to inhibit proliferation and promote apoptosis of HCC cells via the Hippo-Yes-Associated Protein signaling pathway [134]. Likewise, coffee is a very common and widely consumed beverage worldwide. Bioactive molecules in coffee include caffeine, diterpenes, and chlorogenic acid. Coffee has been shown to display anti-fibrotic, anti-neoplastic, anti-inflammatory, and antioxidant effects [135]. It was reported to display a preventive role against the recurrence of HCC after orthotopic liver transplants. Postoperative coffee intake was also found to result in improved overall survival. The anti-HCC effect of caffeine (Figure 13B) was suggested to be mediated by an antagonistic effect on adenosine A2AR-mediated growth of HCC cells. Many natural compounds were described to display anti-HCC activity. S-allylmercaptocysteine derived from garlic has strong hepatoprotective activity. It was shown to exert an anti-HCC effect through the deactivation of MAPK- TGF-β interaction and apoptosis induction in HepG2 cells [136]. This compound was also described to act through an interaction with the cell membrane Wnt-pathway co-receptor [137]. Some alkaloids such as crocin and capsaicin were extensively studied as promising anti-HCC molecules.

#### 4.2.1. Crocin

**Figure 14:**
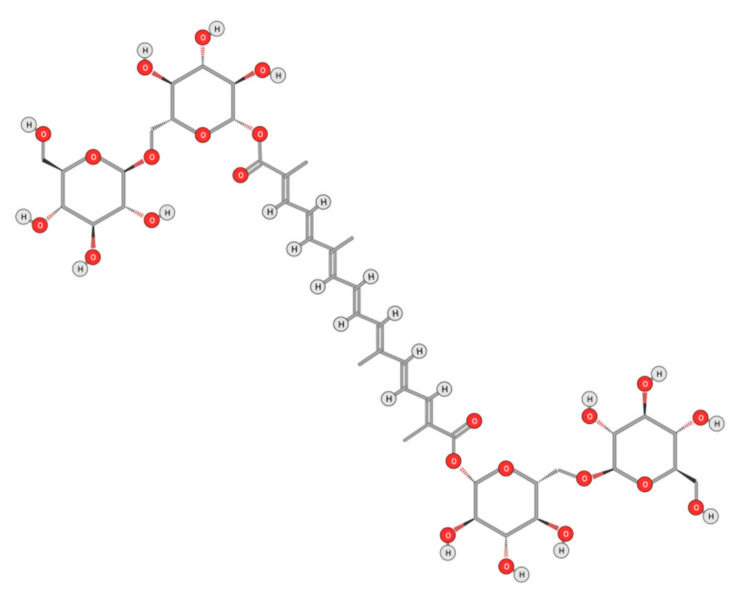
Chemical structure of Crocin.

Crocin (Figure 14), is a major carotenoid responsible for saffron’s color; it was described as a potential anti-HCC drug both alone [104,138] and as a targeted drug encapsulated in nanoparticles [139]). Crocin displayed an anti-liver cancer potential through the inhibition of the IL-6/STAT3 signaling pathways [140]. Upon stimulation with cytokines (IL-6 and IL-11) and growth factors (EGF and PDGF), STAT3 is activated and can be phosphorylated prior to binding to DNA. Crocin inhibits the DNA-binding activity of STAT3 in IL-6-stimulated liver cancer cells. It also induces apoptosis in HCC by inhibiting Akt/mTOR activity [141] and activating Poly ADP-ribose Polymerase 1 (PARP-1) cleavage by caspases-9 and 3 [140]. Furthermore, crocin was able to exert an anti-proliferative effect primarily by inactivating the microtubules’ assembly and dynamics [142]. Most recently, an anti-HCC therapeutic capacity of crocin has been patented [143].

HCC was associated with over expression of telomerase during carcinogenesis to stabilize telomeres thereby enhancing the proliferation of cancer cells [144,145]. The activity of the catalytic subunit of telomerase (hTERT) in HepG2 cells was reported to decrease by 60% upon crocin treatment [144]. Crocin also induces autophagic apoptosis both in HCC, by inhibiting Akt/mTOR activity [141], and in colorectal cancer cells, via autophagy-independent apoptosis [146]. Most recently, crocin’s anti-proliferative effect is reported to be particularly significant in cells with a deficient DNA mismatch repair system [143].

#### 4.2.2. Capsaicin

**Figure 15:**
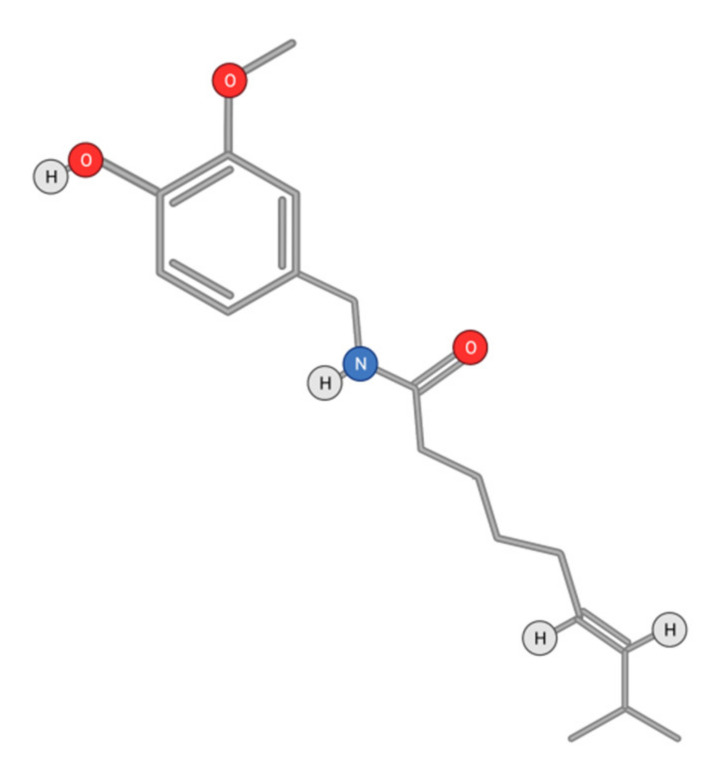
Chemical structure of Capsaicin.

Capsaicin (8-methyl-*N*-vanillyl-6-nonenamide) (Figure 15) is an active alkaloid of chili peppers that has been widely investigated as a therapeutic molecule. Particularly, it was described to display anti-HCC activity [147]. This activity was mainly due to an induction of apoptosis and limitation of cell proliferation [5].

VEGF is produced in HCC cells in concentrations that are correlated with tumor size and disease stage [148]. Capsaicin suppresses VEGF-mediated angiogenesis by reducing the proliferation and differentiation of endothelial cells induced by VEGF and FGF [149].

In conditions with cellular stress or cell damage, cell-cycle arrest can be triggered by activated p53. AMPK plays a major role in tumor development due to its ability to induce p53-mediated cell-cycle arrest [150]. Capsaicin exerts its effects on the transient receptor potential vanilloid 1 (TRPV1), inducing an influx of calcium ions causing cellular activation [151]. Capsaicin activation of AMPK in HepG2 cells via the TRPV1 receptor is triggered by intracellular calcium and calcium/calmodulin-dependent protein kinase beta (CaMKKβ) [152]. Capsaicin-activated AMPK phosphorylates p53 as well as other proteins involved in autophagy and acts as a tumor suppressor [153]. It is noteworthy that capsaicin binding to the TRPV1 receptor activates caspase-3 driven apoptosis in HepG2 cells [154]. The concentrations required to elicit an anti-carcinogenic action are far higher than that needed to activate TRPV1, suggesting additional pathways for capsaicin action [155]. Diversely, the activation of TRPV1 by dietary capsaicin may prevent non-alcoholic fatty liver disease through anti-inflammatory action, as shown on mice liver cells. The capsaicin/TRPV1 receptor binding also stimulated the expression of light chain 3B (LC3-II) and Beclin1, inducing autophagy in HepG2 cells upon peroxisome proliferator-activated receptor (PPARα) activation [156].

The mTOR kinase is involved in multiple essential cellular processes, including cell proliferation, cell survival, and autophagy. mTOR inhibition can activate the formation of autolysosomes, which will degrade the enveloped cellular components, thereby leading to apoptosis [157]. Direct inhibition of the mTOR pathway by capsaicin can induce autophagy [148]. Capsaicin is known to up-regulate the activity of STAT3 via phosphorylation and to induce autophagy in HepG2 cells by triggering the generation of ROS [158]. Furthermore, capsaicin exhibited a chemopreventive role by inhibiting the growth of HCC cells through the induction of apoptosis mediated by a caspase-3-dependent mechanism [159]. In fact, capsaicin induces apoptosis in HepG2 cells by reducing the levels of xIAP and cIAP1 proteins, which are inhibitors of caspase-3 activation [160]. Moreover, capsaicin may induce apoptosis through endoplasmic reticulum (ER), stress, and the subsequent ER release of Ca^2+^. It was also suggested that capsaicin may induce apoptosis in the HepG2 cells by activating a phospholipase C (PLC)-dependent intracellular Ca^2+^ release pathway [161].

Cytochrome c released to the cytosol through the permeabilization of the mitochondrial outer membrane is regulated by B-cell lymphoma 2 (Bcl-2) family proteins. Capsaicin apoptosis induction in HepG2 cells through the release of mitochondrial cytochrome c is dependent on intracellular Ca^2+^ concentrations [160]. Mitochondrial release of cytochrome c leads to an increase in ROS concentrations, with subsequent DNA damage [162,163]. ROS are increased in cancer cells and can promote tumor progression in HepG2 cells, amplifying the rates of invasion and metastasis that can be repelled using antioxidants. Consequently, adequate control of ROS formation can provide a better regulation of disease progression. Oxidative stress may also play an important role in preventing tumor progression through apoptosis. Capsaicin can inhibit the activity of tumor associated NADH oxidase by suppressing the expression of domain transcription factor POU3F2, restricting tumor growth, and inducing apoptosis [158]. Combinatory treatment using capsaicin and sorafenib increased apoptosis through the activation of caspase-9 and poly(ADP-ribose)polymerase [153]. In addition, while sorafenib treatment induces Akt activation, causing a resistance to the treatment, capsaicin inhibits Akt, providing a possible pathway sensitization to sorafenib in HCC cells.

### 4.3. Phytochemicals and Drug Design

Computer aided drug design accelerates the drug discovery process and is based on knowledge of the interacting mechanisms between drugs and target receptors. The structure-based drug design uses information about the receptor binding site to search for appropriate ligands. Virtual Screening is used for computer aided screening of a database (3-dimensional chemical structure databases) of potential ligands based on the highest scores of interactions with the receptor [164]. Many attempts to design anti-cancer agents using phytochemicals were reported. Some examples will be discussed in this report. An in silico approach was used to design anti-HCC therapeutic agents starting from commercial drugs [165]. The target receptor proteins involved in HCC metastasis were BCL-XL and FGF proteins. The receptor was docked to drug analogues and binding energies were estimated. For a better efficiency, the Absorption Distribution Metabolism Excretion Toxicity (ADMET) properties of the drug were improved by ADMET tools. The molecular docking of the analogues with the receptor targets followed by ADMET analysis identified one best candidate for BCL-XL and two best candidates for FGF. Likewise, an in silico analysis of the 9-Octadecenoic acid (Z)-, 2-hydroxy-1-(hydroxymethyl) ethyl ester ligand showed an effective binding energy to Cox-2 receptor [166]. This suggested a mechanism by which this compound prevented liver damage and displayed an anti-carcinogenic potential in DEN induced hepatic carcinoma. Ligand based drug design is used when the target is unknown in order to identify the features of potential receptors (that is, pharmacophore traits). A reverse docking method was applied to identify potential cancer targets for EGCG [167]. Molecular dynamic simulations were then applied to optimize the ligand–receptor interactions. This allowed the identification of 12 signal pathways and 33 target proteins, among which four are novel. These targets and pathways can therefore be used for developing therapeutic strategies. The potential targets of berberine, a natural alkaloid displaying anti-HCC activity, were suggested by pharmacological analysis using databases to carry out a pharmacophore mapping approach [168]. Berberine was suggested to inhibit HCC by affecting the PI3K/Akt signaling pathway. The docking analysis indicated that the binding of berberine to Akt could suppress its activity. This effect was checked on HCC cell lines. Natural compounds displaying anti-HCC anticancer activities often suffer from low bioavailability and selectivity, limiting their therapeutic use [169]. These drawbacks can be reduced through chemical modification or use of a delivery system. A set of methylated(–)-epigallocatechin-3-gallate-4β-triazolopodophyllotoxin derivatives was synthesized and their anti-cancer activity was assayed in vitro. Molecular docking results suggested that one of these compounds displaying an anti-cancer activity had a high binding affinity for epidermal growth factor receptor, a known target for cancer treatment [170]. Recent advances in drug delivery systems describe the use of nanoemulsions, nanoparticles, liposomes, and films to carry various phytochemicals such as berberine, curcumin, resveratrol, camptothecins, and celastrol, showing a promising improved anti-cancer action [169].

## 5. Conclusions

Targeted drugs often work by inhibiting angiogenesis, inducing apoptosis, and blocking cancer cells from proliferating. Targeted therapy has the advantage of delivering powerful suppression of cell development and cancer progression along with lower toxicity to non-malignant cells, which is a common pitfall associated with systemic chemotherapy and radiotherapy. With an increase in our understanding of the molecular biology of HCC, many molecular targets are now associated with HCC genesis and progression. Sorafenib and its derivatives as well as other tyrosine kinase inhibitors have been used in the treatment of HCC. The anti-HCC effect of the bacterial biomolecules such as the macrolide rapamycin and its derivatives have also been demonstrated. miRNAS are widely described as promising anti-HCC biomolecules, able to impair carcinogenesis through control of gene expression and transcription factors. Meanwhile, phytochemicals are regarded as promising natural anti-HCC agents with moderate side effects as compared to chemicals. Phytochemicals include mainly phenolic compounds (such as Decursin and Chlorogenic acid), flavonoids (like quercetin), alkaloids, and carotenoids (such as crocin and capsaicin). The key targets of these anti-HCC biomolecules are mainly peptide growth factor receptors such as EGFR and intracellular signaling proteins involved in cell proliferation and apoptosis. These include the PI3K/Akt/mTOR, JAK/STAT, and Wnt/β-catenin pathways. Angiogenic factors such as VEGF and MMPs are also targeted by these molecules. The anti-HCC effects of these biomolecules can also be exerted through modulating the expression of cell cycle regulators (CDKs), and transcription factors such as NF-κB.

A common feature of phytochemicals is attenuating cancer progression by inhibition of inflammation and induction of apoptosis through caspase-dependent mechanisms or induction of intracellular oxidative stress. The molecular targets and the action mechanisms of these molecules need to be investigated in greater depth. Their efficiency might also be improved by using a structure-based drug design strategy.

## Figures and Tables

**Table 1:** Tyrosine kinase inhibitors under phase II/III clinical studies for HCC treatment [4].

Drug	Targets	Descriptions	Reference
Cediranib 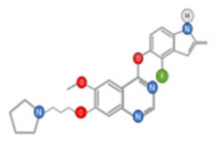	VEGFR	Shows high toxicity and is ineffective for patients with unresectable or metastatic HCC	[30]
Dovitinib 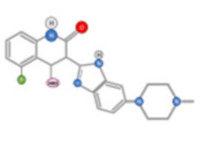	c-KIT, Flt-3, FGFR, VEGFR	Significantly prolongs survival and inhibits primary tumour growth and lung metastasis in HCC xenograft models	[31,32]
Erlotinib 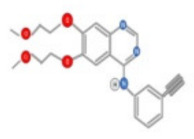	EGFR	Shows modest prolonged progression-free survival and overall survival in patients with unresectable HCC	[33,34]
Gefitinib 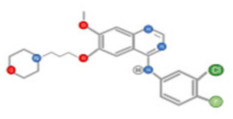	EGFR	Inhibits the tumor growth of HCC xenografts in a mouse model	[35]
Selumetinib 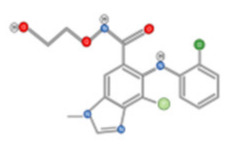	MEK1	Suppresses tumour growth of HCC xenografts in mouse model Shows inadequate antitumour activity with short progression-free survival in patients with locally advanced or metastatic HCC	[36,37]
Brivanib 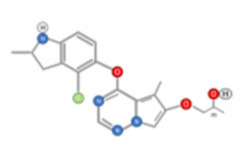	FGFR, VEGFR	Increases apoptosis, reduces microvessel density, and decreases VEGFR phosphorylation Shows promising antitumour activity in patients with advanced HCC	[28,29]
Linifanib 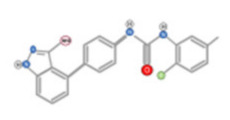	PDGFR, VEGFR	Inhibits tumour growth of HCC xenografts in the mouse model Shows similar overall survival to sorafenib in patients with advanced HCC	[38,39,40]
Sunitinib 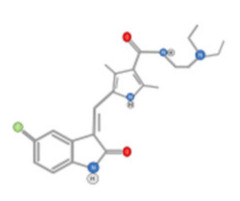	c-Kit, Flt-3, PDGFP, VEGFR	Increases apoptosis and reduces microvessel density of HCC xenografts Displays poor overall survival in patients with advanced HCC and has severe toxicity	[41]
Orantinib 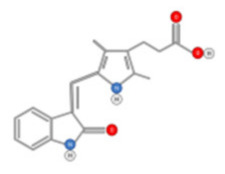	FGFR, PDGFR, VEGFR	Suppresses the tumour growth of subcutaneously co-injected HCC cell line xenografts Shows no improvement in overall survival in patients with unresectable HCC	[42,43]
Bevacizumab	VEGF	Inhibits tumour growth of HCC cell lines or patient-derived HCC xenografts	[44,45]
Cetuximab	EGFR	Shows no obvious response in patients with advanced HCC	[46]

VEGF: Vascular endothelial growth factor; VEGFR: Vascular endothelial growth factor receptor; FGFR: Fibroblast growth factor receptor; EGFR: Epidermal growth factor receptor; MEK1: Mitogen-activated protein kinase (MAPK) kinase; PDGFR: Platelet-derived growth factor receptor; Flt-3: FMS-like tyrosine kinase-3.

**Table 2:** Origins of flavonoids and their targeted signaling pathways that induce apoptosis and inhibit cell proliferation and angiogenesis.

Compound	Main Origin and Structure	Signaling Pathway	HCC Model	Reference
Luteolin 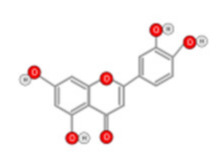	Celery, green pepper, parsley, thyme, dandelion	ROS-mediated pathway Caspase Activation	Hep G2 cells	[114]
Luteolin-7-*O*-glucoside 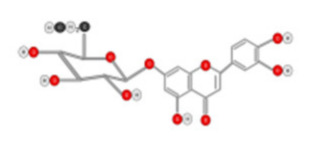	Dandelion, coffee and Cynara scolymus	Arrest G2/M phases of cell by JNK and Caspase activation	HepG2 cells	[115]
Isoorientin 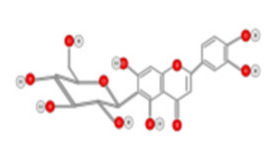	Passion flower, Vitex negundo, Terminalia myriocarpa	Regulation of Cell cycle-related genes ROS-mediated pathway Caspase-3 and caspase-9 activation	Hep G2 cells	[116]
Chrysin 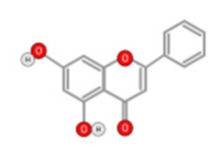	Honey, propolis, passion flowers, and Passiflora caerulea	Downregulation of Skp2 and LRP6 Activation of the p53/Bcl-2/caspase-9	Hep G2 cells	[117]
Oroxylin A 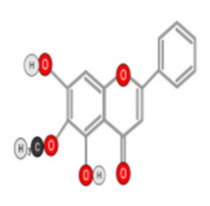	Scutellaria baicalensis and Oroxylum indicum	Suppression of PI3K-PTEN-Akt-mTOR signaling pathway Activation of the ERK-eIF2α-ATF4-CHOP branch of the UPR pathway	HepG2 cells	[118]
Wogonin 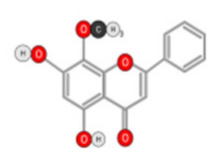	Scutellaria baicalensis	Activation of the UPR pathway and inactivation of Akt signaling	HepG2, SMMC7721, and Hep3B cells	[119]
Baicalein 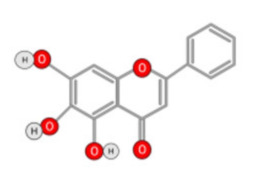	Roots of Scutellaria baicalensis and Scutellaria lateriflora	Inhibition of the PKB/mTOR pathway Blocking MEK-ERK signaling	HepG2 cells Xenograft in mice	[120,121]
Eriodictyol 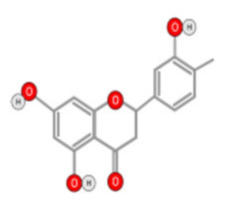	Eriodictyon californicum	Upregulation of Bax and PARP downregulation of Bcl-2	HepG2 cells	[122]
Hesperidin 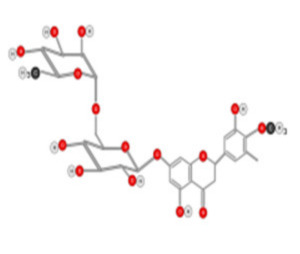	Citrus fruits	Regulation of mitochondrial pathway Death receptor pathway; increases the levels of intracellular ROS, ATP, and Ca^2+^	HepG2 cells Xenograft in mice	[112,123]
Puerarin 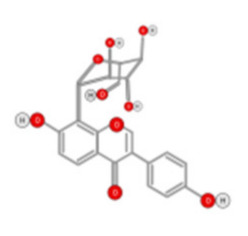	Root of Pueraria	Regulation of MAPK pathways	SMMC-7721 cells	[51]
Galangin 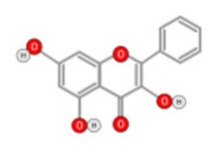	Alpinia officinarum and Helichrysum aureonitens	Pro apoptotic Mitochondrial pathway mediated by Bax	HepG2, HepG2, Hep3B, and PLC/PRF/5 cells	[124,125]
Epigallocatechin gallate 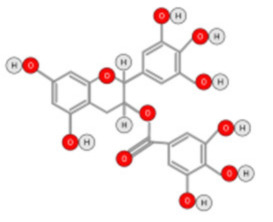	Tea leaves	Inhibition of tyrosine kinase receptors Downregulation of PI3K/Akt activity; downregulating Bcl-2 alpha and Bcl-xl by inactivation of NF-κB Hypoxia	SMMC7721, SKhep1, HLE, HepG2, HuH-7, PLC/PRF/5 cells; Xenograft in mice	[110,126]
Dihydromyricetin 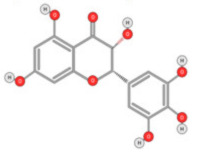	Ampelopsis japonica, Hovenia dulcis	Reduction of TGFβ via p53- dependent pathway	HepG2 cells	[127]
Kurarinol 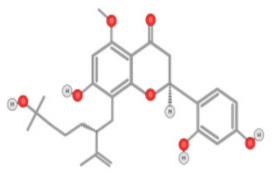	Roots of Sophora flavescens	Suppressing STAT3 signaling	HepG2, Huh-7, and H22 cells; Xenograft in mice	[113]
Eriocitrin 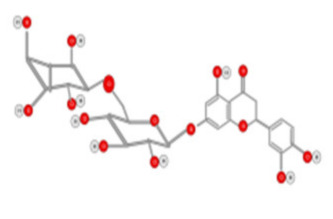	Lemon fruits	Upregulation of p53, cyclin A, cyclin D3, and CDK6 through activation of mitochondrial pathway	HepG2 cells	[128]
Fisetin 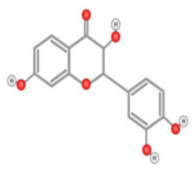	Strawberries, apples, persimmons, onions, and cucumbers	Regulation of CDK5 signaling NRF2-mediated oxidative stress response Glucocorticoid signaling ERK/MAPK signaling	HepG2 cells	[129]
Kaempferol 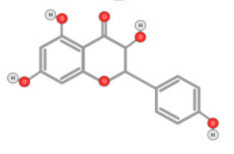	kale, beans, tea, spinach, and broccoli.	Inhibition of MAPK and HIF-1	Huh7 cells	[130]
Theaflavins 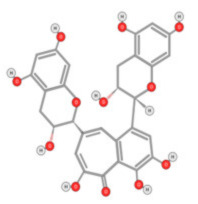	Black tea	Activating the caspase pathwayBlockage of STAT3 pathway	HepG2 cells Xenograft in mice	[111]

## Data Availability

Not applicable.
